# Development and Field Validation of WaziSense, a Low-Cost Solar-Powered IoT Smart Tensiometer for Soil–Water Monitoring and Irrigation Scheduling in Semi-Arid Agriculture

**DOI:** 10.3390/s26175348

**Published:** 2026-08-24

**Authors:** Hassine Ben Abdallah, Liliya Naui, Mourad Bakri, Felix Markwordt, Mohamed Abdur Rahim, Corentin Dupont, Mohamed Ali Ben Abdallah, Mourad Rezig

**Affiliations:** 1 National Institute for Research in Rural Engineering, Water and Forestry (INRGREF), University of Carthage, Ariana 2080, Tunisia; hassine.benabdallah@iresa.agrinet.tn (H.B.A.); mouradbakri1919@gmail.com (M.B.); benabdallah_medali@yahoo.fr (M.A.B.A.); rezigue_mourad@yahoo.fr (M.R.); 2 Waziup e.V., Räcknitzhöhe 50/52, 01217 Dresden, Germany; felix.markwordt@waziup.org (F.M.); abdur.rahim@waziup.org (M.A.R.); corentin.dupont@waziup.org (C.D.)

**Keywords:** soil-water monitoring, irrigation scheduling, Agriculture 4.0, Internet of Things, smart tensiometer, WaziSense, soil matric potential, LoRa, automated machine learning (AutoML), irrigation forecasting, deficit irrigation, irrigation scheduling, semi-arid

## Abstract

Water scarcity in semi-arid regions makes efficient irrigation scheduling a priority, yet farm-level adoption of soil-moisture monitoring remains limited by the cost, low portability and installation complexity of commercial sensing systems. This study presents the development and field validation of WaziSense, a low-cost, solar-powered Internet-of-Things (IoT) smart tensiometer, developed within the OSIRRIS platform for soil-water monitoring and irrigation scheduling. The device couples a Watermark granular-matrix sensor and a DS18B20 temperature probe to an ATmega328P microcontroller (Arduino Pro-Mini, 3.3 V, 8 MHz) with long-range LoRa communication and a maximum-power-point-tracking (MPPT) solar-charging stage, logging soil matric potential and soil temperature every 15 min. An open-source edge/cloud stack (WaziGate, WaziApp) retrieves weather forecasts from an open API and runs an automated machine learning (AutoML) regression pipeline that forecasts soil-water dynamics and the time to a user-defined threshold, from which irrigation is scheduled and its applied volume verified by a flow meter. The system was deployed at three bioclimatic sites in Tunisia (durum wheat at Cherfech, citrus at Nabeul, apple at Sbeitla), with tensiometers installed at 20 and 40 cm depths, and validated against commercial 10HS capacitive probes coupled to a ZL6 data logger, with which the co-located readings were significantly correlated (r = 0.81). Calibrated readings showed a strong relationship between soil–water content and soil–water potential (R^2^ = 0.99), and the edge forecasting model reproduced soil–water dynamics on unseen data (Sbeitla apple site, 5-day horizon) with R^2^ = 0.73, RMSE = 0.35, MAE = 0.23 and MPE = 12.52%. With a material cost under about 90 EUR per node and fully open-source hardware and software, WaziSense is one to two orders of magnitude cheaper than commercial monitoring stations, offering an affordable, reproducible and scalable tool for data-driven irrigation in water-limited agriculture.

## 1. Introduction

Agriculture is the largest consumer of freshwater worldwide, accounting for roughly 70% of global withdrawals, and this share is even higher in the arid and semi-arid regions of the Mediterranean, where climate change is intensifying droughts and competition for water [1,2]. Raising water use efficiency at the farm scale has therefore become a central objective of sustainable and digital agriculture, and the paradigms of Agriculture 4.0 and 5.0 place data-driven, sensor-based decision-making at the core of this transition [3,4]. Matching irrigation to the actual soil-water status, rather than to fixed calendars, is one of the most effective ways to achieve this, provided that reliable, affordable and easy-to-deploy soil sensing is available [1,5].

A wide range of soil-moisture sensors are commercially available, yet few growers use them to schedule irrigation, mainly because of their cost, limited portability and installation complexity [1,5]. Granular-matrix (Watermark) tensiometers are particularly attractive for this purpose: they relate directly to the soil matric potential, they are inexpensive and are robust in the field, and, when combined with low-power IoT connectivity, they can bring continuous root zone monitoring within reach of smallholders [6].

Several IoT-based irrigation systems have been proposed. Wireless-sensor-network systems automate irrigation but often lack integration with weather forecasting or rain detection [7,8]; GSM-based controllers enable remote management but depend on manual input and network availability [9]; and cloud-centric systems improve decision-making but require stable connectivity and paid subscriptions that are not always feasible for small farms [10]. Coupling in situ sensing with weather forecasts and machine learning has been shown to reduce water use and improve scheduling [6,7], and low-cost IoT tensiometers have been developed and validated [11]. Within the same OSIRRIS framework, the present system has already supported deficit irrigation studies in apple [12], winter wheat [13] and Washington Navel orange [14]; here, we focus on the development, full disclosure and field validation of the sensing-and-decision device itself.

The aim of this work is to develop and validate an affordable, energy-autonomous and reproducible IoT tensiometer for soil-water monitoring and irrigation scheduling in semi-arid agriculture. Specifically, the objectives are (i) to design a low-cost, solar-powered LoRa sensing node from widely available components; (ii) to describe its embedded firmware, on-device calibration and open-source edge/cloud software, including the machine learning forecasting pipeline and the irrigation-scheduling logic, in a fully reproducible way; (iii) to validate the device in the field, at three bioclimatic sites and against a commercial 10HS/ZL6 reference; and (iv) to quantify its material cost relative to commercial monitoring stations.

## 2. Materials and Methods

### 2.1. The OSIRRIS Platform and the WaziSense Smart Tensiometer

WaziSense was developed within the OSIRRIS project (Precision Irrigation with Cost-effective and Autonomic IoT at the Edge), a TUNGER 2+2 bilateral collaboration between Germany (Waziup e.V., Dresden, and INNOTEC21) and Tunisia (INRGREF and INRAT, Menzah, Tunisia), funded by the German Federal Ministry of Education and Research (BMBF) and the Tunisian Ministry of Higher Education and Scientific Research (2022–2025) [13]. It is an affordable, easily deployable IoT/edge-computing platform. The smart tensiometer integrates LoRa wireless communication, an MPPT solar charge controller and an ATmega328P microcontroller (Microchip Technology, Chandler, AZ, USA) programmed in C/C++. It comprises a Watermark tensiometer (Irrometer Company, Inc., Riverside, CA, USA) and a temperature probe connected to Arduino Pro-Mini (Arduino, Ivrea, Italy) operating at 3.3 V and 8 MHz; soil matric potential and soil temperature are logged automatically every 15 min and transmitted over LoRaWAN to the WaziGate gateway, which stores the data, synchronizes them with the OSIRRIS cloud and hosts the edge applications (WaziApp). The WaziAct module drives the pump for automated irrigation.

The platform was developed iteratively. A first prototype (Prototype 1) was built and tested in the laboratory in 2022 and deployed with the WaziGate platform at the three experimental sites for calibration and validation in 2023. A more robust prototype (Prototype 2) was then evaluated in the field in 2024 under controlled deficit irrigation (CDI) and partial root zone drying (PRD); a fully automated prototype (Prototype 3), in which the smart tensiometer drives the irrigation controller, was subsequently tested.

**Figure 1:**
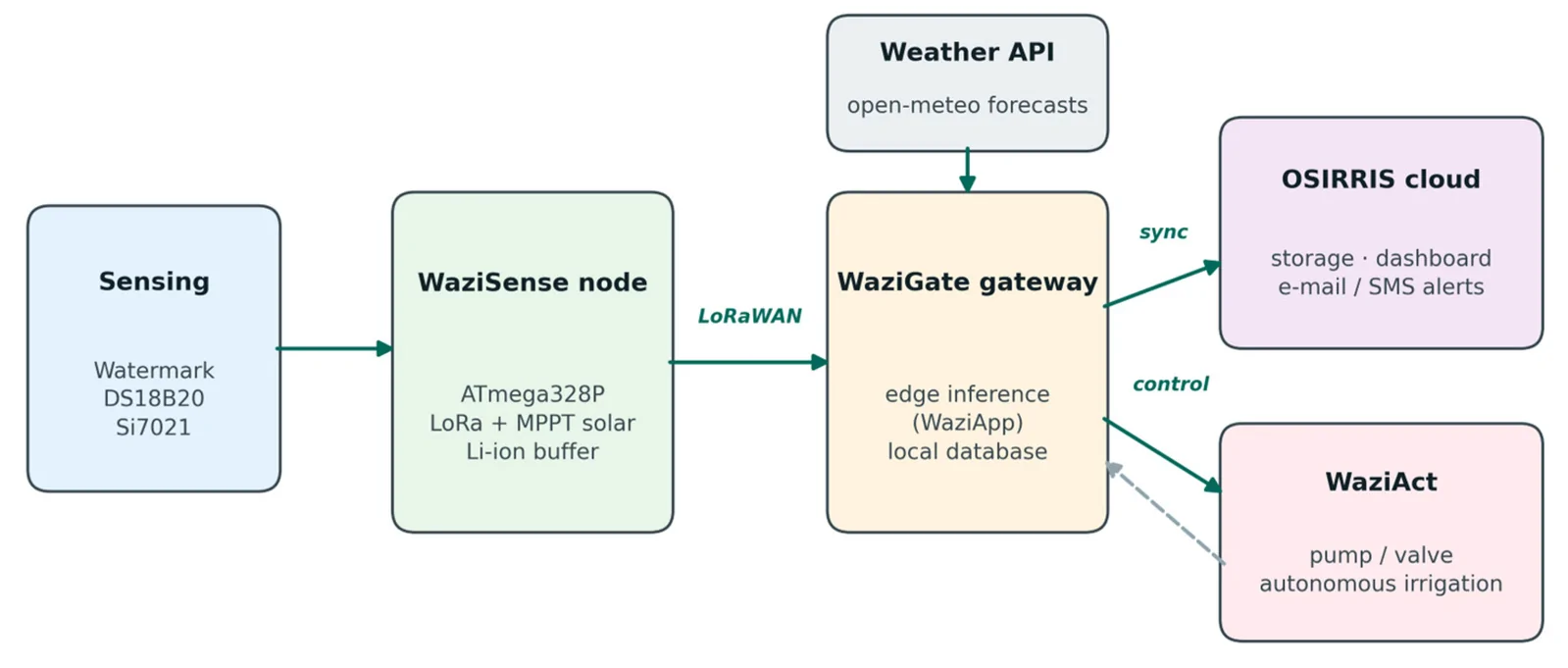
Architecture of the OSIRRIS platform: the WaziSense node acquires soil and air data and transmits them over LoRaWAN to the WaziGate gateway, which performs edge inference (WaziApp), stores the data and synchronizes with the OSIRRIS cloud; weather forecasts from an open API feed the machine learning model, and the WaziAct module drives the pump for automated irrigation. Solid arrows show the data and command flow; the dashed arrow shows the soil-response feedback from WaziAct to the gateway. Box colours only group the platform layers and have no quantitative meaning.

The overall architecture of the platform is shown in Figure 1.

### 2.2. Hardware Architecture

**Figure 2:**
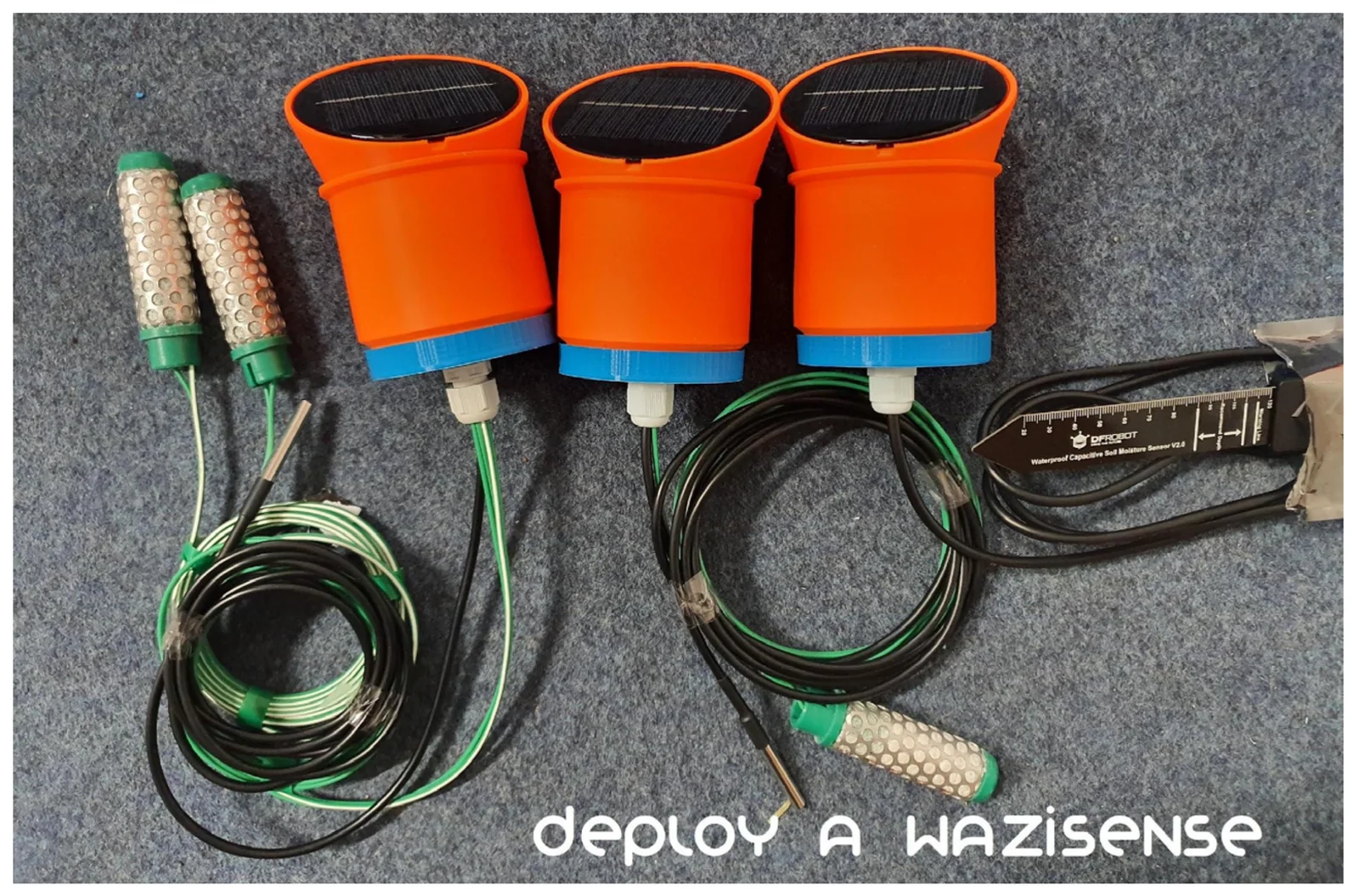
WaziSense nodes, showing the 3D-printed solar enclosures, the Watermark granular-matrix sensors and the temperature probes.

The sensor node is a production-ready LoRa node built around the ATmega328P microcontroller (Arduino Pro-Mini 3.3 V 8 MHz bootloader). It can operate as a battery-less node harvesting energy from the environment, using a super-capacitor circuit able to retain energy for multiple transmissions; in the most recent test, more than 100 messages were transmitted at spreading factor 12 on a single full charge of the super-capacitors. The node (Figure 2) combines LoRa communication, an MPPT solar charge controller and the ATmega328P microcontroller. The selected sensing element is the Watermark granular-matrix tensiometer; combining the soil temperature measured by a waterproof DS18B20 sensor (Maxim Integrated, San Jose, CA, USA) with the Watermark resistance allows the soil matric potential to be computed in centibars. The node also embeds a Si7021 sensor (Silicon Labs, Austin, TX, USA) that measures air temperature and relative humidity over the I2C bus, and exposes terminals for external probes, so that it can host either a Watermark tensiometer or a capacitive soil-moisture probe.

### 2.3. Power System and Energy Autonomy

**Figure 3:**
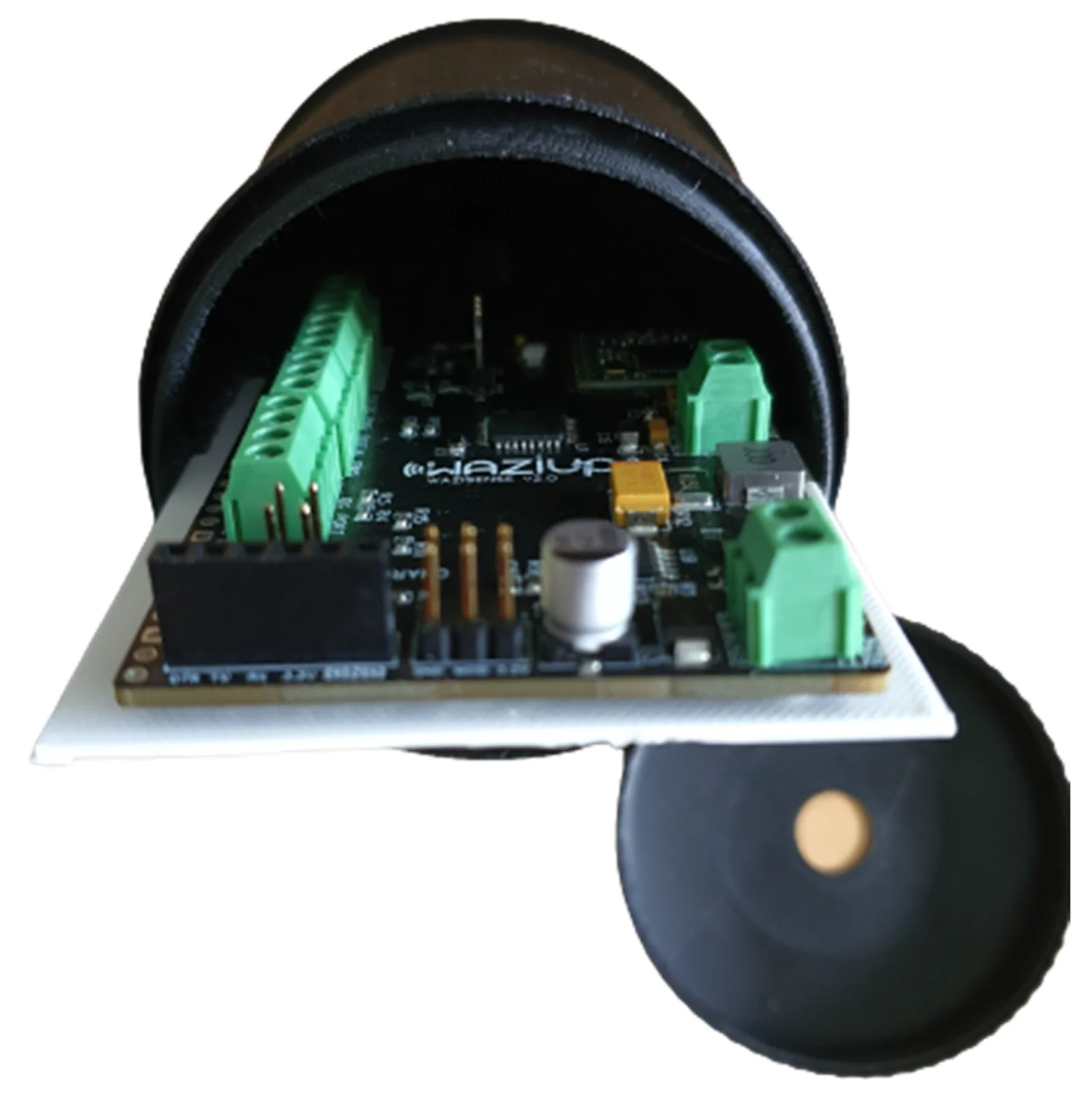
WaziSense electronics mounted on the sliding sledge, partly extracted from the 3D-printed ASA enclosure with its bottom service cover.

In deep-sleep mode at 4 V, the node draws only 2.5 mA (about 10 mWh) and accepts supply voltages from 3.3 to 16 V. For short-term data collection, a 4-cell AA battery holder is connected directly to the node; for long-term operation, solar energy is harvested through a 6 V MPPT solar charge controller that charges a 3.7 V Li-ion cell up to 4.2 V and provides short-circuit and overcharge protection. The 6 V/1 W panel recharges the buffer cell in roughly 9 h under optimal conditions. AA batteries alone last about two months, while the lower consumption of the second-generation node extends this autonomy to about six months. The enclosure is 3D-printed in UV-resistant ASA to IP55 (Figure 3), and a 75 mm PVC pipe serves as a protective cover, mast and cable conduit. The two super-capacitors store about 0.041 Wh, whereas the 2500 mAh Li-ion buffer stores about 10 Wh, roughly 250 times more, which guarantees continuous operation through cloudy periods.

### 2.4. Gateway and Embedded Software

**Figure 4:**
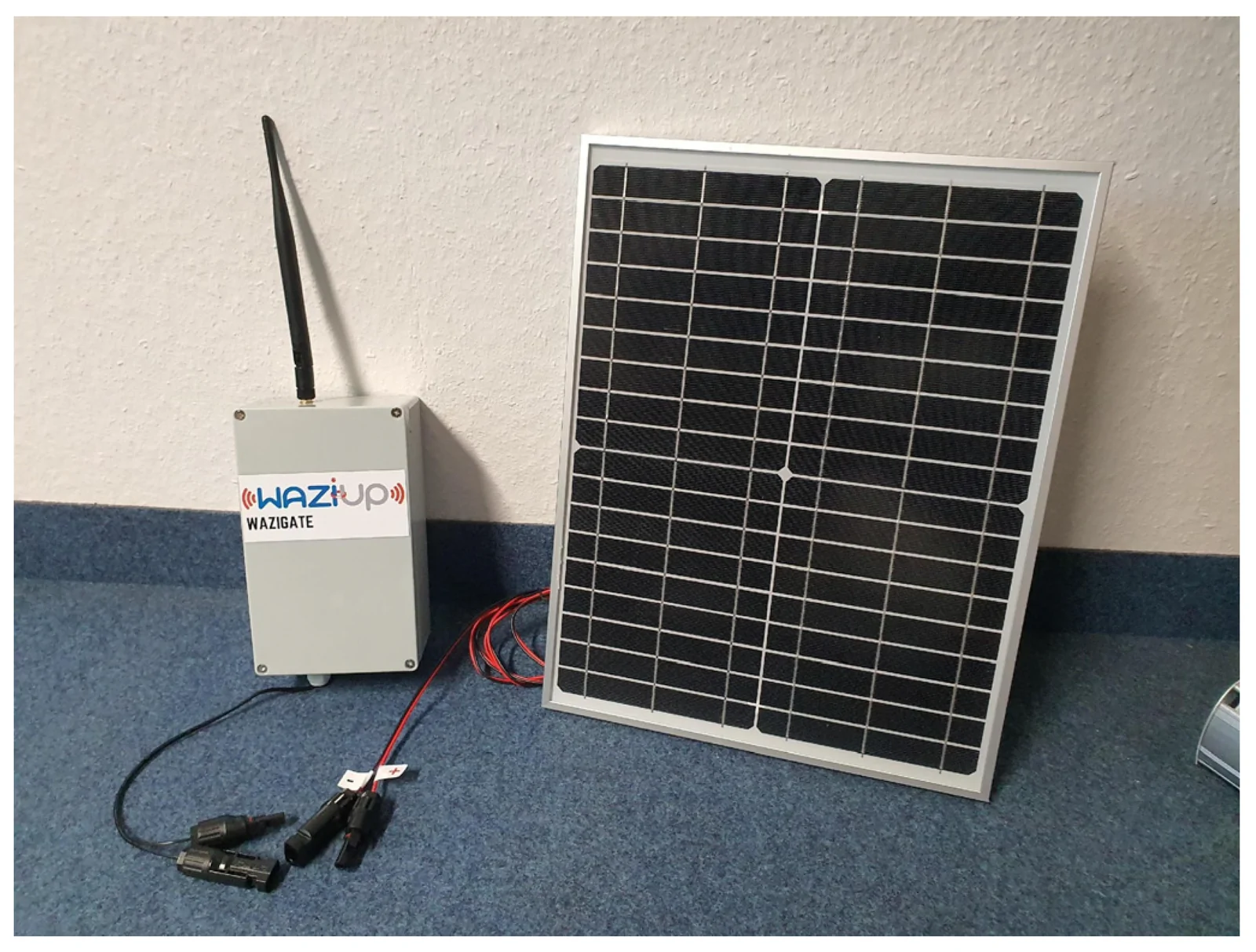
WaziGate IoT LoRa gateway with its solar panel.

The WaziGate IoT LoRa gateway (Figure 4) connects up to 100 sensor and actuator nodes and hosts edge applications directly on the device. It offers a LoRa range of up to 10–12 km and a WiFi hotspot for connectivity, and adapts to WiFi, 3G and Ethernet. Its embedded database and web-based visualization module enable remote management even without Internet access.

At the core of WaziSense, the embedded firmware orchestrates five tasks:

- Measurement execution: Sensor activation, data acquisition and conversion of raw readings into meaningful values;
- Calibration: Conversion of the raw readings into calibrated centibar values;
- LoRa data transmission: Formatting into LoRa packets and reliable transfer to the gateway;
- Energy management: Monitoring of the battery voltage and application of energy-saving measures;
- Low-battery adaptation: Lengthening of the transmission interval when the battery is low.

### 2.5. Edge Machine Learning Forecasting Model

Soil-water forecasting is performed by the open-source WaziApp “irrigation-prediction” application, which runs on the WaziGate at the edge; its full source code is publicly available under an Apache-2.0 license [15], making the forecasting component entirely reproducible. The pipeline is built on the PyCaret automated machine learning (AutoML) regression library [16] and proceeds as follows. Historical and forecast weather variables for the plot’s GPS location are retrieved from the open-meteo API (https://open-meteo.com; air temperature, relative humidity, precipitation, cloud cover, shortwave radiation, wind speed and direction, reference evapotranspiration, and soil temperature and moisture at several depths); these are combined with the in situ soil-tension and temperature series and with the recorded irrigation events. A rolling mean of the grouped soil-moisture signal is used as the prediction target.

The dataset is split while preserving temporal order and evaluated with a sliding-window (time-series) cross-validation. The forecasting relies on a standard, openly available AutoML framework (PyCaret [16]) and is not, in itself, a machine learning contribution of this work; the novelty of the study lies in the integration, low-cost implementation and field validation of the complete IoT sensing-and-decision platform. Using PyCaret’s compare_models routine, sixteen candidate regressors are trained and ranked by the coefficient of determination (linear, ridge, lasso, elastic-net, Bayesian-ridge and Huber regressions, k-nearest neighbors, decision tree, random forest, extra trees, AdaBoost, gradient boosting, XGBoost, LightGBM and CatBoost). Because no single algorithm performed best across the five test periods evaluated, the deployed predictor is not a single fixed model but an ensemble: the best-performing candidates are combined by blending and stacking, and the resulting stacked ensemble is tuned over predefined grids, finalized and deployed for edge inference. The main tuned hyper-parameters are the regularization strength and the intercept for the linear, ridge, lasso and elastic-net regressions; the number of trees (100–500), the maximum depth (up to 20) and the minimum samples per split and per leaf for the random-forest and extra-trees models; the number of trees, the learning rate (0.01–0.1), the maximum depth (2–7) and the sub-sampling ratio for the gradient-boosting, XGBoost, LightGBM and CatBoost models; the number of neighbors (3–11) for k-nearest neighbors; and the hidden-layer size and learning-rate schedule for the multilayer perceptron. The complete hyper-parameter grids are provided in the open-source code [15]. The implementation uses Python (version 3.9) with PyCaret, scikit-learn, XGBoost, CatBoost and TensorFlow/Keras (software environment and versions in the open-source code [15]), and the full design, model list and development are documented in the public OSIRRIS deliverables D3.1 and D3.2 [17], making the component fully reproducible. Model performance on unseen data is reported with the mean absolute error (MAE), root-mean-square error (RMSE), mean percentage error (MPE) and coefficient of determination (R^2^) (Section 3.5), and the day-before-irrigation recommendations were compared with the irrigation actually performed and reviewed by agronomic experts.

### 2.6. Irrigation Scheduling and Control Logic

Irrigation is scheduled from the forecast rather than from a fixed calendar. Following the FAO-56 soil-water-balance approach, root zone depletion is tracked and irrigation is planned so as to keep it between field capacity and the readily available water (RAW) threshold, the root zone being replenished towards field capacity after each event; deficit treatments apply a reduction coefficient to the crop evapotranspiration (ETc) [13,14]. Operationally, WaziApp forecasts the time at which the soil signal will cross a user-defined threshold and issues an irrigation recommendation; the corresponding water amount is applied through the WaziAct actuator, and the volume actually delivered is confirmed by a flow meter, with a tolerance of 20% and a verification window of about three hours. The stop condition is therefore the delivery of the computed, flow-meter-verified water amount that returns the root zone to the target soil-water status, rather than a fixed irrigation duration.

### 2.7. Experimental Sites and Deployment

The platform was tested and validated under Tunisian farming practices across three bioclimatic stages. Site 1 is the INRGREF experimental station at Cherfech, Grand Tunis (durum wheat). Site 2 is the INRGREF station at Oued Souhil, Nabeul (mean annual rainfall about 440 mm), on 27-year-old Washington Navel orange trees (*Citrus sinensis* L. Osbeck). Site 3 is the CFPA apple orchard at Sbeitla, Kasserine (525 m a.s.l.; semi-arid, mean rainfall about 254.8 mm), on the cultivars Galaxy and Richared. At each site, the WaziSense tensiometers were installed in the root zone at 20 and 40 cm depths, about 30 cm from the plant, consistent with the 0–40 cm root zone monitored in the related field trials [12,13]; the node exposes terminals for several probes, so additional depths (e.g., 60 cm) can be monitored where a deeper root zone requires it. Gravimetric soil sampling was used to establish the soil-water retention relationship. Two complementary sources of weather data were used. Daily meteorological variables recorded by a standard agro-meteorological station located within each experimental unit were used to compute reference evapotranspiration (ET_0_) with the FAO-56 Penman–Monteith equation and hence meet the crop water requirements of the trials [13,14]. Independently, the edge forecasting model does not require an on-site station: it retrieves historical and forecast weather variables for the plot GPS location from the open-meteo API, which keeps the deployed system low-cost and usable anywhere. Commercial 10HS capacitive probes coupled to a ZL6 data logger (METER Group, Pullman, WA, USA) were first used to build the training database; from December 2022, the WaziSense prototypes and the WaziGate platform were operated at the three sites under controlled deficit irrigation and partial root zone drying.

## 3. Results

### 3.1. Soil Matric Potential and Volumetric Water Content Dynamics

**Figure 5:**
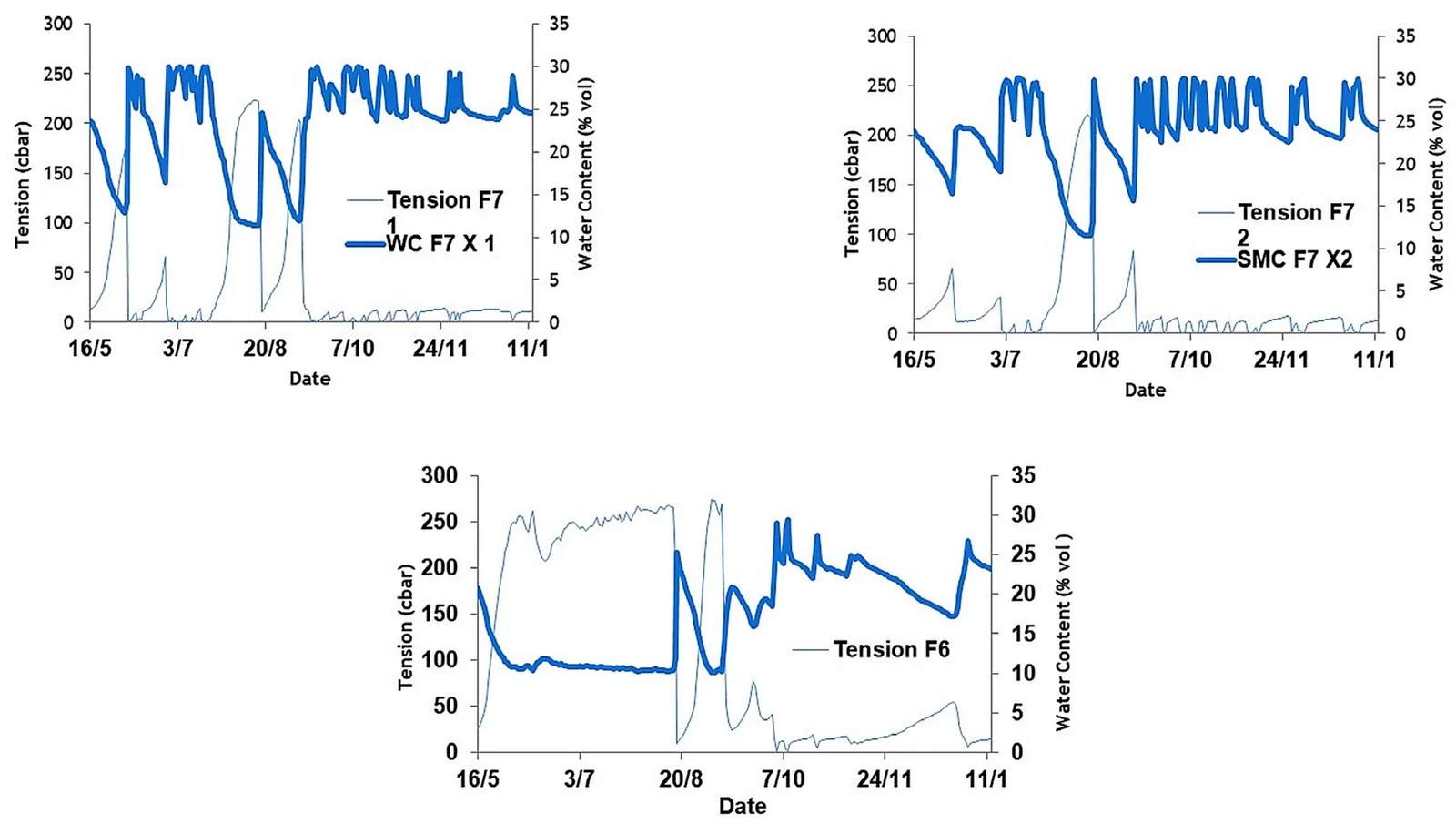
Temporal variations in soil matric potential (cbar) and volumetric soil-water content (% vol.) measured with WaziSense (Prototype 2). Positions 1 and 2 denote the two monitored sensor locations; the blue line is the volumetric soil-water content.

Figure 5 shows the temporal variations in soil matric potential (cbar) and volumetric soil-water content (% vol.) measured with WaziSense (Prototype 2). The device tracked the wetting and drying cycles of the root zone continuously and in real time, capturing the expected inverse relationship between matric potential and water content over the monitored period.

### 3.2. Calibration and Reliability of the Readings

**Figure 6:**
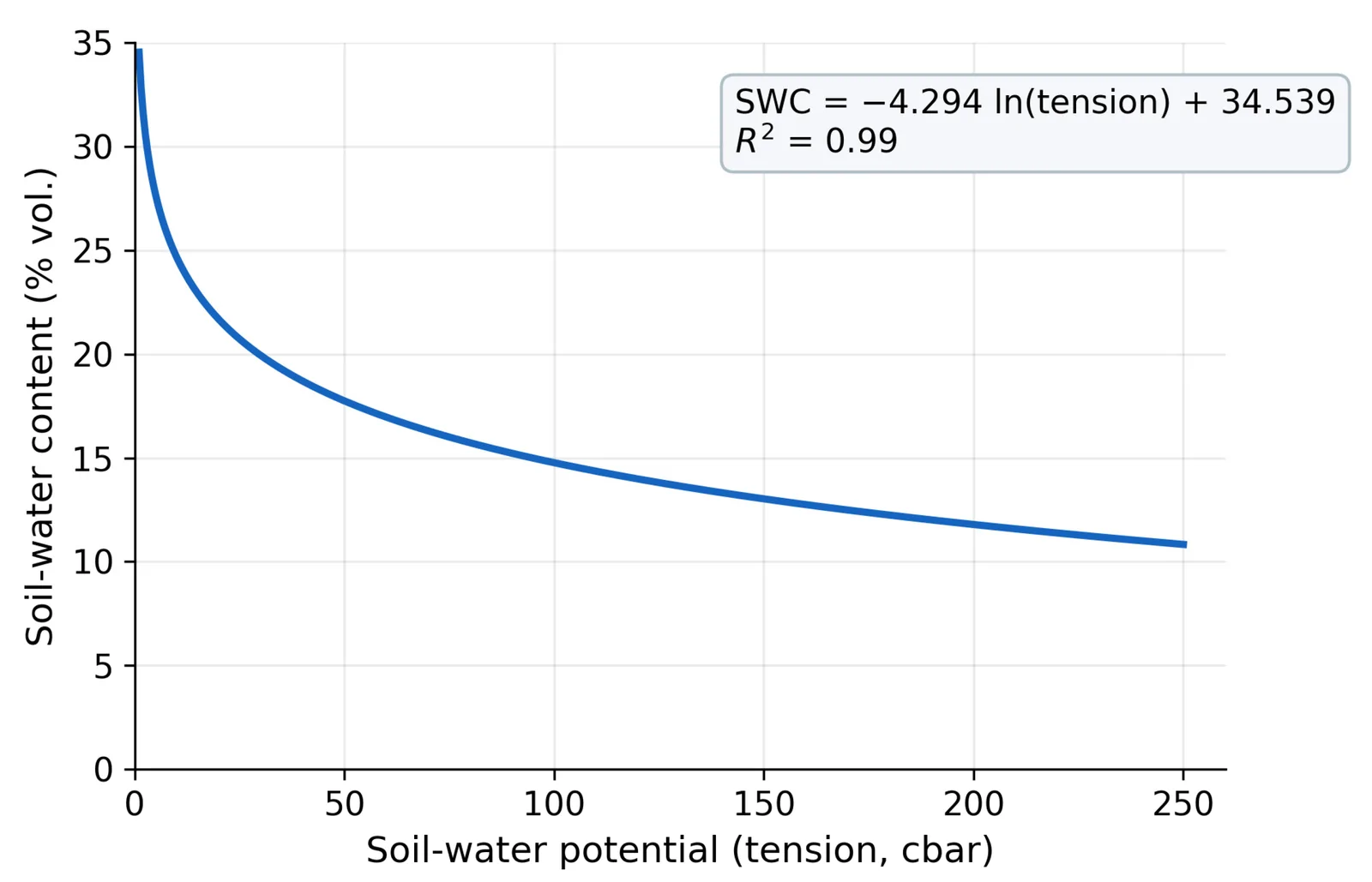
Calibration curve relating volumetric soil-water content (% vol.) to soil-water potential (cbar) (2023–2024; R^2^ = 0.99).

Figure 6 presents the relationship between soil-water content (% vol.) and soil-water potential (cbar) measured with WaziSense during the 2023–2024 cropping season. A strong logarithmic relationship was obtained (SWC = −4.294 ln(tension) + 34.539), with a coefficient of determination of R^2^ = 0.99, confirming the reliability and internal consistency of the calibrated readings.

### 3.3. Daily Root Zone Water Depletion

**Figure 7:**
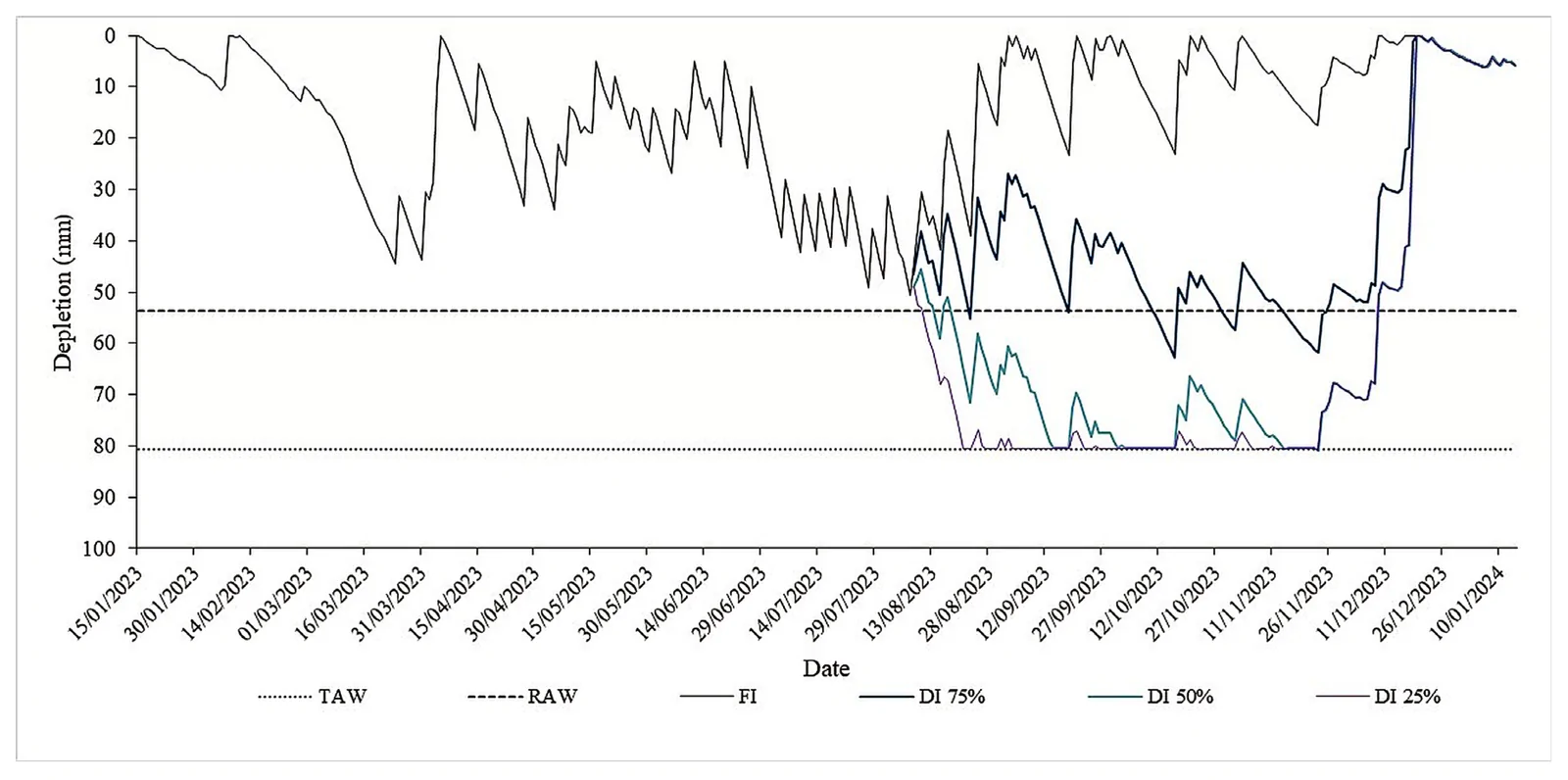
Daily patterns of root zone water depletion (mm) under full irrigation (FI) and deficit irrigation (DI-75%, DI-50%, DI-25% ETc.), 2023–2024 (TAW: total available water, 80 mm; RAW: readily available water, 54 mm). Adapted from [14].

Figure 7 shows the daily dynamics of water depletion (mm) in the root zone under full irrigation (FI) and deficit irrigation at 75%, 50% and 25% of ETc. WaziSense resolved the day-to-day depletion relative to the total available water (TAW) and readily available water (RAW) thresholds, showing how the deficit treatments progressively crossed the RAW threshold while the full-irrigation treatment remained within the readily available range, information that directly supports the timing of irrigation.

### 3.4. Validation Against a Commercial Reference Probe

**Figure 8:**
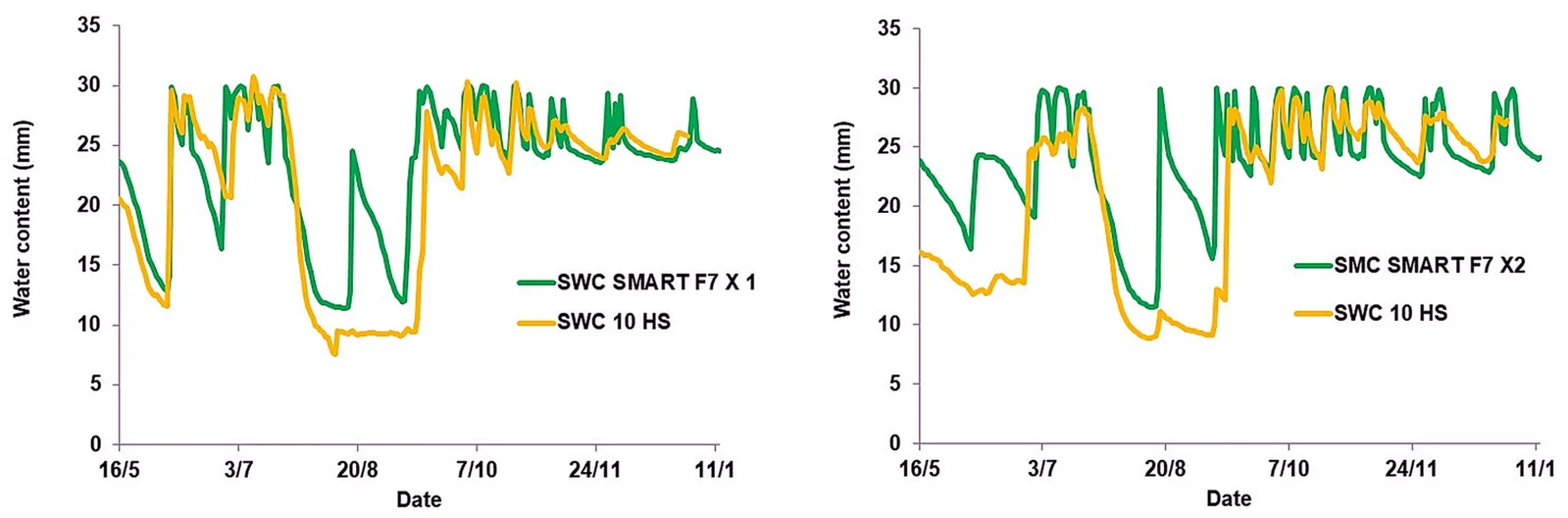
Soil-water content (mm) measured simultaneously by the commercial 10HS/ZL6 reference probe and by WaziSense (Prototype 2) at two sensor positions.

**Figure 9:**
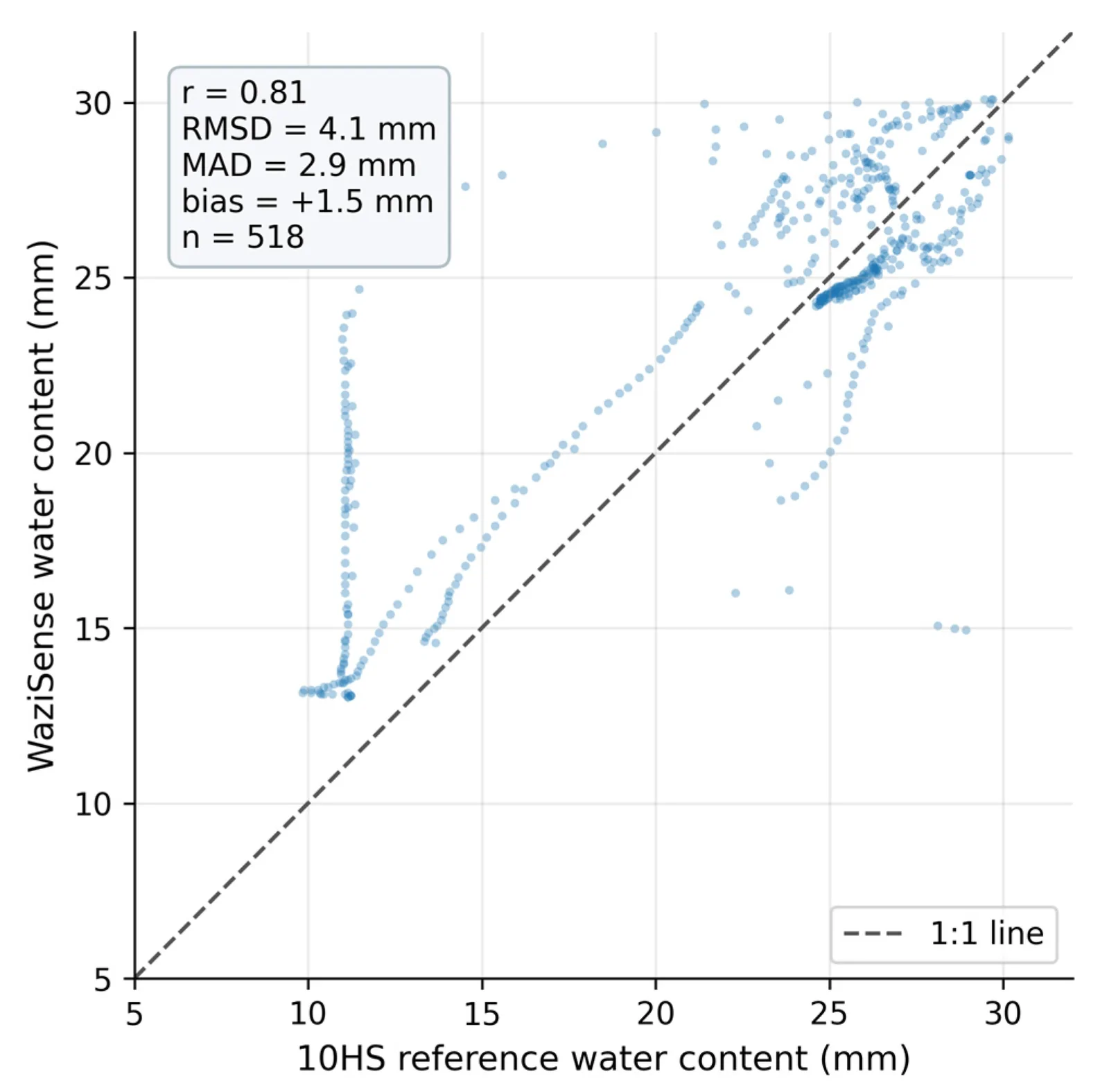
Scatter plot of the water content measured by WaziSense against the commercial 10HS/ZL6 reference over the monitoring period, with the 1:1 line and the agreement statistics (r: correlation coefficient; RMSD: root-mean-square difference; MAD: mean absolute difference; n: number of paired observations).

The WaziSense readings were validated against commercial 10HS capacitive probes coupled to a ZL6 data logger, installed alongside the smart tensiometers at 20 and 40 cm depths. Figure 8 shows the soil-water content measured simultaneously by the 10HS reference and by WaziSense (Prototype 2): the two instruments followed the same wetting and drying dynamics closely throughout the monitoring period, confirming that the low-cost device reproduces the soil-water status given by an established commercial system. A quantitative comparison of the two co-located series over the monitoring period showed that they were significantly correlated (correlation coefficient r = 0.81), with a root-mean-square difference of the order of 4 mm, a mean absolute difference of about 2.9 mm and a small positive bias (the WaziSense reading was, on average, about 1.5 mm wetter than the 10HS reference) (Figure 9).

### 3.5. Edge Forecasting Performance

The retained stacked-ensemble model was evaluated on unseen data at the Sbeitla apple site for a 5-day forecast horizon, with the rolling-mean soil-moisture signal as a target. The performance metrics on the test set are summarized in Table 1: the model reproduced the soil-water dynamics with a coefficient of determination of R^2^ = 0.73, an RMSE of 0.35, an MAE of 0.23 and an MPE of 12.52%, providing actionable forecasts of soil-water depletion for irrigation scheduling.

### 3.6. Energy Autonomy and Communication

Operating at 2.5 mA in deep-sleep mode (about 10 mWh) and harvesting energy through the MPPT solar stage, WaziSense maintained continuous 15 min logging throughout the monitoring period. On AA batteries alone, autonomy increased from about two months for the first-generation node to about six months for the second-generation node, and the 6 V/1 W panel recharged the buffer cell in about 9 h under optimal conditions. The super-capacitor variant transmitted more than 100 messages at spreading factor 12 on a single charge, and the WaziGate gateway provided a LoRa range of up to 10–12 km, ensuring reliable communication across the three sites.

### 3.7. Cost Analysis

At representative component prices, the material cost of a WaziSense node is under 90 EUR, in agreement with the figure reported for the same device in the related field studies [12]; the itemized procurement record used here (Table 2), which includes import costs, totals about 110 EUR. In either case, the node is comparable to previously reported low-cost prototypes (76 USD [11]; about 72 EUR [18]) and one to two orders of magnitude below commercial soil-moisture telemetry stations, the price of which ranges from several hundred to several thousand Euros. The shared WaziGate gateway adds about 74 EUR and serves up to 100 nodes.

## 4. Discussion

The strong agreement between soil-water content and soil-water potential (R^2^ = 0.99) confirms that the calibrated WaziSense provides internally consistent readings of the soil-water status, in line with granular-matrix sensors and previous low-cost IoT tensiometers [11]. The close agreement with the commercial 10HS/ZL6 reference (Figure 8) shows that the low-cost device reproduces the soil-water dynamics of an established system, while the edge AutoML model anticipated those dynamics on unseen data with R^2^ = 0.73 and RMSE = 0.35, consistent with the range reported for IoT-based irrigation models [8,10].

Triggering irrigation from the soil-water status and from a forecast of threshold crossing is a well-established and effective approach [10,11]. It nevertheless has a known limitation: because root zone moisture is not uniformly distributed, soil-moisture-only control can lead either to salt accumulation or to water waste. More advanced, crop-water-demand-based strategies have already been demonstrated in practice. In particular, Kocian et al. base irrigation control on crop water demand rather than on a fixed soil-moisture threshold: they combine a dynamic Bayesian-network prediction of the crop coefficient (crop evapotranspiration) with automated lysimetry to regulate the irrigation duration through a target leaching fraction, quantitatively reducing irrigation water use and runoff under field conditions [19]; comparisons of soil-moisture- and weather-based scheduling with other advanced IoT irrigation-management approaches have likewise been reported [20,21]. Compared with these methods, the advantages of WaziSense are its very low cost (under 90 EUR per node), its energy autonomy and its fully open hardware and software, which make the whole sensing-and-decision chain affordable and reproducible. Its main limitations, relative to the weighing-lysimeter approach, are that it derives crop water requirements from the FAO-56 water balance using standard, tabulated crop coefficients rather than from a dynamically predicted crop coefficient, and that it does not measure drainage or regulate the leaching fraction. Integrating crop-coefficient prediction and leaching-fraction-based control into the WaziSense platform is therefore a clear and worthwhile next step. Positioned within the Agriculture 4.0/5.0 framework [3,4], the main strengths of the present device are its very low cost, its energy autonomy, and, importantly, the full openness of both hardware and software, which make the whole sensing-and-decision chain reproducible and lower the barrier to large-scale, data-driven irrigation.

Three design choices further differentiate WaziSense from earlier work. First, the edge-computing architecture and on-device calibration reduce the dependence on permanent connectivity and paid cloud subscriptions, which have previously limited cloud-centric systems [10]. Second, the MPPT solar stage and the lower consumption of the second-generation node extend the maintenance-free autonomy from about two to about six months, addressing the portability and installation constraints that hinder sensor adoption [2]. Third, the use of widely available components keeps the node cost under about 90 EUR (Table 2 and reference [12]), a prerequisite for large-scale uptake. Validated across three bioclimatic sites and three crops (durum wheat, citrus and apple), the device already underpins the agronomic outcomes reported within the OSIRRIS framework [12,13,14]. Remaining limitations include the single-season scope of the forecasting evaluation and the need to consolidate the metrological comparison over additional seasons and depths.

## 5. Conclusions

This work presented WaziSense, a low-cost, solar-powered IoT smart tensiometer with an edge forecasting model, developed and validated within the OSIRRIS platform at three bioclimatic sites in Tunisia. The device delivered reliable, real-time monitoring of soil matric potential and volumetric water content (calibration R^2^ = 0.99), agreed closely with a commercial 10HS/ZL6 reference at 20 and 40 cm depth, and forecasted soil-water dynamics on unseen data with R^2^ = 0.73 using a fully open-source AutoML pipeline. Combining an edge-computing architecture, solar-powered autonomy of up to six months, open hardware and software, and a material cost of under about 90 EUR per node, WaziSense offers an affordable, reproducible and scalable tool for soil-water monitoring and irrigation scheduling in water-limited agriculture. Future work will extend the multi-season validation, integrate weighing- and leaching-fraction-based control, and quantify the water savings achievable under operational farm conditions.

## Figures and Tables

**Table 1:** Performance of the retained edge machine learning model on unseen test data (target: rolling-mean soil-moisture signal).

Metric	Value
Mean absolute error (MAE)	0.23
Root-mean-square error (RMSE)	0.35
Mean percentage error (MPE)	12.52%
Coefficient of determination (R^2^)	0.73

**Table 2:** Bill of materials of a WaziSense node and the shared WaziGate gateway (actual project procurement, OSIRRIS, 2022), and cost comparison with commercial soil-moisture monitoring solutions.

Item	Unit Cost (EUR)	Source
WaziSense node (this work):		
ATmega328P/WaziAct board	12.00	[a]
Antenna	0.50	[a]
Wires and jumpers	0.10	[a]
Watermark 200SS soil-moisture sensor	59.90	[a]
DS18B20 soil-temperature sensor	1.70	[a]
Waterproof casing (IP55)	5.99	[a]
2-way switch	0.20	[a]
AA battery holder (4-cell)	1.20	[a]
AA batteries (4 × 0.37)	1.48	[a]
MPPT solar charge controller	5.39	[a]
Solar panel (6 V, 1 W)	5.20	[a]
Li-ion battery (2500 mAh, 3.7 V)	14.60	[a]
LoRa head	2.00	[a]
WaziSense node—total	~110	[a]
WaziGate gateway (shared, up to 100 nodes):		
Raspberry Pi 3B+ (Raspberry Pi Foundation, Cambridge, UK) + power supply + case	~71	[a]
LoRa head + antenna	2.50	[a]
WaziGate gateway—total	~74	[a]
Commercial solutions:		
METER 10HS + ZL6 cloud logger (reference)	~600–850 + 170–200/sensor	[b]
Sentek TriScan 90 cm probe (Sentek Technologies, Stepney, SA, Australia)	~2115	[c]
Metos iMETOS station (Pessl Instruments, Weiz, Austria)	~3240	[c]

Sources: [a] INRGREF/OSIRRIS project procurement record (2022); [b] METER Group (list prices on request); [c] NSW DPI AgTech catalog. Indicative values; EUR/USD parity assumed for the order-of-magnitude comparison.

## Data Availability

The data presented in this study are available on request from the corresponding author. The source code of the forecasting application is openly available at https://github.com/Waziup/waziup.irrigation-prediction (accessed on 1 July 2026).

## Abbreviations

The following abbreviations are used in this manuscript:

API	Application Programming Interface
ASA	Acrylonitrile Styrene Acrylate
AutoML	Automated Machine Learning
CDI	Controlled Deficit Irrigation
ET_0_	Reference Evapotranspiration
ETc	Crop Evapotranspiration
FAO-56	FAO Irrigation and Drainage Paper 56
IoT	Internet of Things
IP55	Ingress Protection rating 55
LoRa	Long Range (radio modulation)
LoRaWAN	Long Range Wide Area Network
MAE	Mean Absolute Error
ML	Machine Learning
MPE	Mean Percentage Error
MPPT	Maximum Power Point Tracking
OSIRRIS	Precision Irrigation with Cost-effective and Autonomic IoT at the Edge (project)
PRD	Partial Root-zone Drying
RAW	Readily Available Water
RMSE	Root Mean Square Error
SWC	Soil Water Content
TAW	Total Available Water
WaziAct	Waziup actuation module
WaziApp	Waziup edge application
WaziGate	Waziup IoT LoRa gateway
WaziSense	Waziup smart-tensiometer sensor node
